# Association between multimorbidity patterns and incident depression among older adults in Taiwan: the role of social participation

**DOI:** 10.1186/s12877-023-03868-4

**Published:** 2023-03-27

**Authors:** Hsin-En Ho, Chih-Jung Yeh, James Cheng-Chung Wei, Wei-Min Chu, Meng-Chih Lee

**Affiliations:** 1 Institute of Medicine, Chung Shan Medical University, Taichung, Taiwan; 2 Department of Family Medicine, Taichung Armed Forces General Hospital, Taichung, Taiwan; 3 School of Medicine, National Defense Medical Center, Taipei, Taiwan; 4 School of Public Health, Chung-Shan Medical University, Taichung, Taiwan; 5 Department of Allergy, Immunology & Rheumatology, Chung Shan Medical University Hospital, Taichung, Taiwan; 6 Graduate Institute of Integrated Medicine, China Medical University, Taichung, Taiwan; 7 Department of Family Medicine, Taichung Veterans General Hospital, Taichung, Taiwan; 8 School of Medicine, National Yang Ming Chiao Tung University, Taipei, Taiwan; 9 Department of Post - Baccalaureate Medicine, College of Medicine, National Chung Hsing University, Taichung, Taiwan; 10 Research Center for Geriatrics and Gerontology , National Chung Hsing University, Taichung, Taiwan; 11 Department of Epidemiology of Aging , National Center for Geriatrics and Gerontology, Obu, Japan; 12 Department of Family Medicine, Taichung Hospital, Ministry of Health and Welfare, No. 199, Sec. 1, Sanmin Rd., West Dist, Taichung City, 403 Taiwan (R.O.C.); 13 Institute of Population Health Sciences, National Health Research Institutes, Miaoli County, Taiwan; 14 College of Management, Chaoyang University of Technology, Taichung, Taiwan; 15 Study Group of Integrated Health and Social Care Project, Ministry of Health and Welfare, Taichung, Taiwan

**Keywords:** Multimorbidity patterns, Depression, Social participation, Elderly adults

## Abstract

**Background:**

Previous research has found different multimorbidity patterns that negatively affects health outcomes of older adults. However, there is scarce evidence, especially on the role of social participation in the association between multimorbidity patterns and depression. Our study aimed to explore the relationship between multimorbidity patterns and depression among older adults in Taiwan, including the social participation effect on the different multimorbidity patterns.

**Methods:**

Data were retracted from the Taiwan longitudinal study on ageing (TLSA) for this population-based cohort study. 1,975 older adults (age > 50) were included and were followed up from 1996 to 2011. We used latent class analysis to determine participants’ multimorbidity patterns in 1996, whereas their incident depression was determined in 2011 by CES-D. Multivariable logistic regression was used to analyse the relationship between multimorbidity patterns and depression.

**Results:**

The participants’ average age was 62.1 years in 1996. Four multimorbidity patterns were discovered through latent class analysis, as follows: (1) Cardiometabolic group (n = 93), (2) Arthritis-cataract group (n = 105), (3) Multimorbidity group (n = 128) and (4) Relatively healthy group (n = 1649). Greater risk of incident depression was found among participants in the Multimorbidity group (OR: 1.62; 95% CI: 1.02–2.58) than the Relatively healthy group after the multivariable analysis. Compare to participants in the relatively healthy group with social participation, participants in the arthritis-cataract group without social participation (OR: 2.22, 95% CI: 1.03–4.78) and the multimorbidity group without social participation (OR: 2.21, 95% CI: 1.14–4.30) had significantly increased risk of having depression.

**Conclusion:**

Distinct multimorbidity patterns among older adults in Taiwan are linked with the incident depression during later life, and social participation functioned as a protective factor.

**Supplementary Information:**

The online version contains supplementary material available at 10.1186/s12877-023-03868-4.

## Background

We are living in an ageing world. In 2020, the United Nations estimated approximately 727 million persons aged ≥ 65 years, which is projected to grow continuously to more than double by 2050. [1] Additionally, the global population proportion over 60 years will almost double from 12 to 22% between 2015 and 2050. [2] This global ageing wave is changing healthcare systems and the manner of seeking better health in old age. [3] Currently, non-communicable diseases are the principal cause of death, leading to a significant disease burden and years lived with disability (YLD). [4].

Multimorbidity is among the major concerns among aged people. It refers to patients with more than two or three chronic diseases simultaneously. [5] Researchers have discovered that multimorbidity has increased the disease burden and medical costs over decades. [6] It also possesses several negative impacts on health and geriatric syndromes, such as falls, [7] being institutionalised, [8] major adverse kidney events, [9] frailty, [10] disability [11] and even mortality. [12, 13]

Different patterns/clusters of multimorbidity in different populations have recently been discovered. [14] Hence, it is important to understand the composition of each multimorbidity pattern to study the aetiology of common diseases. Furthermore, different multimorbidity patterns with different characteristics were discovered in different countries to cause different health consequences. For example, Yao et al. found that people in China aged ≥ 50 years had primarily four multimorbidity patterns: (1) a vascular-metabolic group, (2) a stomach-arthritis group, (3) a cognitive-emotional group and (4) a hepatorenal group. [15] A previous study revealed that cardiometabolic multimorbidity of the four multimorbidity patterns in Taiwanese older people was linked with increased disability and mortality. [16] Furthermore, Zheng et al. showed that complex cardiometabolic multimorbidity pattern among five multimorbidity patterns was linked with higher mortality in the United States. [17].

Depression among the elderly is another crucial issue in an older population. [18] Clinically significant depressive symptoms are present in approximately 15% of community-dwelling older adults. [19] Also, depression among the elderly causes great suffering with adverse effects on health, such as suicide-related ideation, [20] social isolation, [21] frailty, [22] disability [23] and mortality. [24].

Multimorbidity has been associated with depression. A systematic review showed that depression was two to three times more likely in people with multimorbidity than in those without multimorbidity or with no chronic physical condition. [25] Additionally, older adults with multimorbidity are more likely to have depression, anxiety and stress symptoms. [26] A study in China showed that multimorbidity was linked with elevated functional limitations and depression among people older than 45 years. [27] Another study in the UK revealed that multimorbidity and depression were strongly associated among female UK Biobank participants with a previous breast cancer diagnosis. [28].

Conversely, social participation is an important promoter of physical and mental health among the elderly. Social participation includes social connections, informal social participation and volunteering. [29] Previous research disclosed that the elderly with certain social participation patterns had less chance of developing physical disability. [30] Also, there was a negative association between social participation and depression in older adults. Liu et al. discovered that older adults who maintained or increased social participation had reduced depressive symptoms, whereas individuals with decreased social participation reported elevated depressive symptoms. [31].

However, social participation’s role in different multimorbidity patterns associated with depression remains unexplored. Moreover, no study concerning this question has been performed. Therefore, understanding the answers would help healthcare professionals rapidly identify the high-risk population and take measures to prevent depression. Therefore, we analysed the relationships between multimorbidity patterns and depression among older adults in Taiwan in this 16-year, population-based cohort study.

## Methods

### Data source and study groups

The data were extracted from a population-based, nationally-representative study, Taiwan Longitudinal Study on Aging (TLSA), initiated in 1989 by the Taiwanese Bureau of Health Promotion and the Population Studies Center of the Institute of Gerontology at the University of Michigan, USA. Data were collected from systematically selected representative samples of the Taiwanese population, including institutionalised older adults. Respondents were longitudinally followed at every three to four years intervals. Highly trained interviewers performed all personal interviews with careful supervision to maintain quality. Nine study waves have been initiated, including 1989, 1993, 1996, 1999, 2003, 2007, 2011, 2015 and 2019. The current study used 1996 and 2011 data, excluding individuals with depression in 1996. The 10-item CES-D measures depressive symptoms. The scores on this scale ranged from 0 to 30, with ten or more as the cut-off point for depression. We also excluded participants who died or loss follow-up during the observation period. Detail of the study was shown in our previous work. [32].

This study is a retrospective study. The Institutional Review Board of Health Promotion Administration, Ministry of Health and Welfare approved this study. The approval number was BHP-2007-002.

### Independent variable

The multimorbidity patterns in 1996 were analysed by assessing 12 chronic conditions, including hypertension, diabetes mellitus, coronary artery disease, stroke, cancer, lung disease, arthritis or rheumatic disease, hepatobiliary disease, renal disease (including stone), gout, hip fracture and cataract. Additionally, participants were asked, ‘Have you ever had the disease of …?’ If they answered ‘No’ or ‘I don’t know’, they were classified as a disease-free group.

Another important independent variable is social participation. Participants who had engaged in paid (Participants were asked:” Are you currently in a job? Or not in a job?”) or voluntary work (Participants were asked:” Are you involved in any voluntary social service work?”) or had participated in community activities (Participants were asked:” Are you currently engaged in community activities, such as clubs of singing, dancing club, Tai Chi or karaoke?”) in 1996 were considered to have experienced social participation.

### Dependent variables

Depressive symptoms were assessed using the 10-item CES-D in the seventh wave (2011), [33, 34] a modified version of the original 20-item full-length CES-D. [35] Adequate reliability and validity have been demonstrated for the Chinese version of the 10-item CES-D among the Chinese elderly. [36] Ten depressive symptoms were examined, including (1) poor appetite; (2) bad mood; (3) everything was an effort; (4) could not sleep well; (5) lonely; (6) people were unfriendly; (7) could not get going; (8) sad; (9) happy and (10) life was good. Each respondent was asked if they had any among the ten depressive symptoms within the last seven days. A four-point scale was used for each item, indicating how often one experienced a given symptom (0 = never, 1 = rarely, 2 = sometimes, 3 = often or chronically). The positive effect of items was reverse-coded. The score ranges from 0 to 30, while a higher score indicates more severe depressive symptoms. We adopted a score of ten or higher as the cut-off point proved to have high sensitivity (0.85) and specificity (0.80), indicating clinically significant depression. [36].

### Confounding variables

We recorded and analysed the following variables in the third wave (1996): age, sex, income level, social participation, self-rated health, health behaviour (smoking, drinking, betel nut chewing and exercise habits), disability and admission.

The income level was determined by asking, ‘Are you satisfied with your income?’ The answers were categorised as ‘good’ (very satisfied/satisfied), ‘fair’, or ‘poor’ (unsatisfied/very unsatisfied). Additionally, self-rated health was evaluated for each individual and categorised as ‘good’ (very good/good), ‘fair’, or ‘poor’ (poor/very poor). Furthermore, participants’ reported exercise habits were classified into ‘no exercise,’ ‘≤ 2 times’, ‘3–5 times’ and ‘≥ 6 times’ per week. Functional status is the ability to perform the activities of daily living (ADL), such as bathing, dressing, eating, getting out of bed, walking and using the bathroom. [37] Respondents were also defined as ‘with disability’ if they had difficulty in one of the six ADLs.

### Statistical analysis

We used latent class analysis (LCA) to estimate the disease patterns. Both models (lower Akaike information criterion or Bayesian information criterion values) fit and interpretability were used to select the most appropriate model. Demographic and clinical characteristics were descriptively analysed for each group. Continuous variables were assessed using analysis of variance and the chi-square test to assess categorical variables. Univariate and multivariable logistic regression analyses explored the relationships between disease patterns and depression. Test of interaction between distinct multimorbidity patterns and social participation was also performed by multivariable logistic regression. The SAS procedure PROC LCA 1.3.2 (https://www.latentclassanalysis.com/software/proc-lca-proc-lta) was used to perform LCA. All data were analysed using SAS version 9.4 software (SAS Institute, Cary, NC, USA). Statistical significance was considered at *p* < 0.05.

## Results

**Fig. 1:**
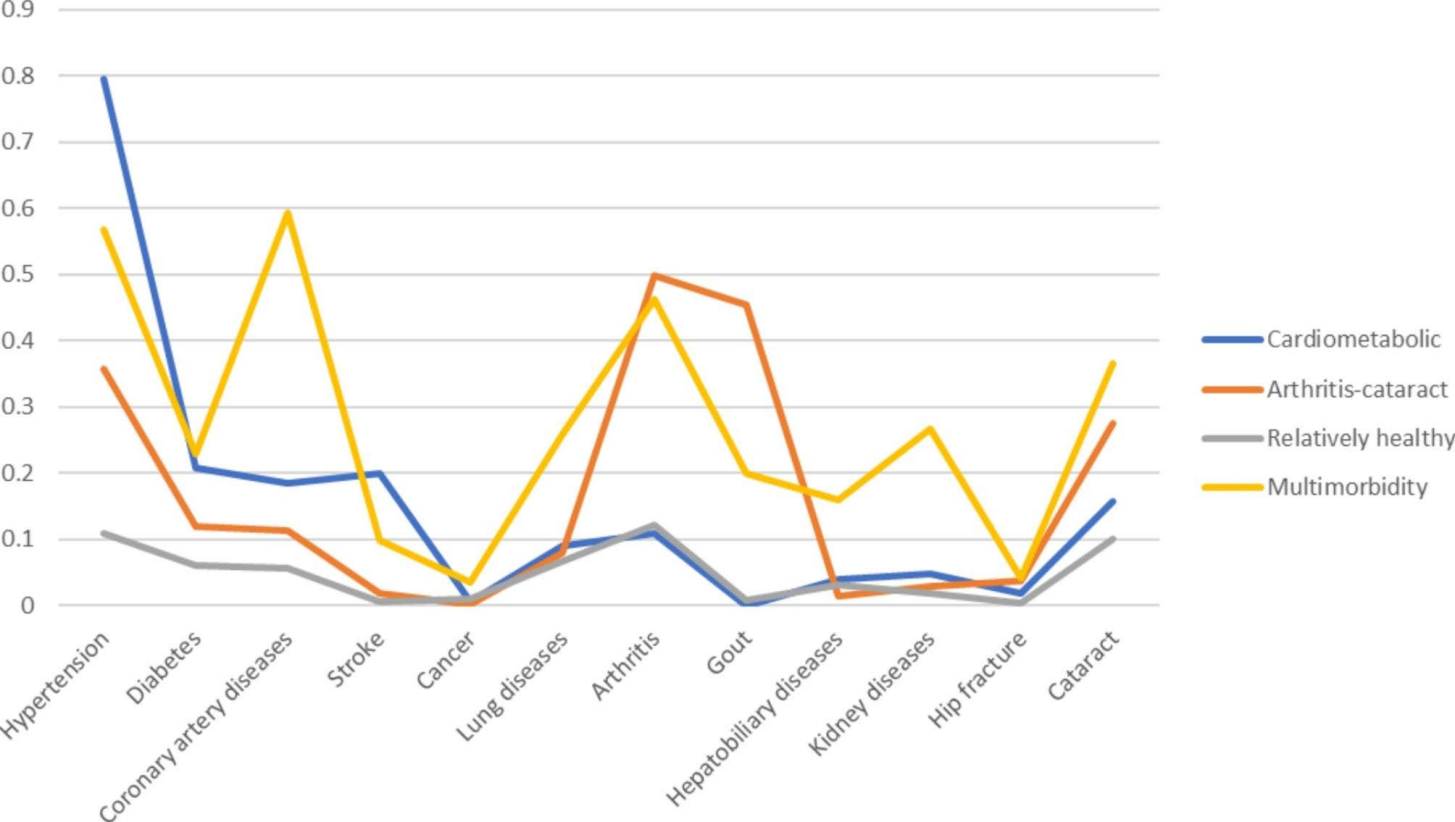
Multimorbidity patterns by latent class analysis in 1996

Table 1 shows the demographic and clinical characteristics of the participants in 1996. The participants with numbers of 1975 were enrolled in the final analysis with a mean age of 62.1 years. The LCA developed four groups and five group of multimorbidity patterns with AIC: 948.34 and 938.41, BIC: 1270.27 and 1342.40, adjusted BIC: 1108.21 and 1139.04, respectively). Therefore, we chose four groups of multimorbidity patterns which include the cardiometabolic group (n = 93), arthritis-cataract group (n = 105), relatively healthy group (n = 1649) and multimorbidity group (n = 128) (Fig. 1). The age distribution, gender, social participation, self-rated health, smoking, alcohol consumption, betel nut chewing, exercise, disability and admission in the past year differed between the groups after the statistical analysis. For example, there were more females in the cardiometabolic and multimorbidity group, whereas males predominated among all participants.

**Table 1:** Demographic and clinical characteristics of the participants in 1996

					Multimorbidity Patterns			
	Characteristics		Total	Cardiometabolic	Arthritis-cataract	Relatively healthy	Multimorbidity	*P-value*
			1975	n = 93	n = 105	n = 1649	n = 128	
Age			62.1(7.6)	64.1(7.1)	64.3(7.9)	61.5(7.6)	66(6.7)	< 0.0001
Sex								
	Male		1013(51.3%)	42(45.2%)	54(51.4%)	868(52.6%)	49(38.3%)	0.0104
	Female		962(48.7%)	51(54.8%)	51(48.6%)	781(47.4%)	79(61.7%)	
Income satisfaction								0.2146
	Poor		222(11.8%)	7(18.1%)	10(10.0%)	193(12.3%)	12(9.6%)	
	Fair		851(45.1%)	48(55.8%)	48(48%)	706(44.8%)	49(39.2%)	
	Good		814(43.1%)	31(36.1%)	42(42%)	677(42.9%)	64(51.2%)	
Social participation								0.0093
	Yes		1300(65.8%)	51(54.8%)	64(60.9%)	1111(67.4%)	74(57.8%)	
	No		675(34.2%)	42(45.2%)	41(39.1%)	538(32.6%)	54(42.2%)	
Self-rated health								< 0.0001
	Poor		312(16.4%)	23(26.7%)	27(26.7%)	203(12.8%)	59(47.2%)	
	Fair		677(35.7%)	37(43%)	44(43.6%)	552(34.8%)	44(35.2%)	
	Good		908(47.9%)	26(30.2%)	30(29.7%)	830(52.4%)	22(17.6%)	
Smoking								0.0102
	Yes		471(23.8%)	15(16.1%)	24(22.9%)	414(25.1%)	18(16.1%)	
	No		1503(76.2%)	78(83.9%)	81(77.1%)	1235(74.9%)	110(85.9%)	
Alcohol consumption								0.002
	Yes		485(246%)	14(15.1%)	19(18.1%)	432(26.2%)	20(15.6%)	
	No		1489(75.4%)	79(84.9%)	86(81.9%)	1216(73.8%)	108(84.4%)	
Betal nut chewing								0.0126
	Yes		141(7.1%)	8(8.6%)	14(13.3%)	116(7%)	3(2.3%)	
	No		1834(92.9%)	85(91.4%)	91(86.7%)	1533(93%)	125(97.7%)	
Admission in the past year								< 0.0001
	Yes		194(9.8%)	12(12.9%)	21(20%)	134(8.1%)	27(21.1%)	
	No		1781(90.2%)	81(87.1%)	84(80%)	1515(91.9%)	101(78.9%)	
Exercise								0.0044
	No		902(45.7%)	34(36.6%)	47(44.8%)	774(46.9%)	47(36.7%)	
	≦ 2times/Week		126(6.4%)	4(4.3%)	10(9.5%)	107(6.5%)	5(3.9%)	
	3–5 times/Week		175(8.9%)	5(5.4%)	6(5.7%)	155(9.4%)	9(7%)	
	≧ 6times/Week		772(39.1%)	50(53.8%)	42(40%)	613(37.2%)	67(52.3%)	
Disability								0.0008
	Yes		73(3.7%)	8(8.6%)	9(8.6%)	49(3%)	7(5.5%)	
	No		1902(96.3%)	85(91.4%)	96(91.4%)	1600(97%)	121(94.5%)	

Data in tables are numbers (%) for categorical variables and means (SD) for continuous variables

Table 2 presents the univariate logistic regression that explored the relationship between each characteristic and incident depression. Different multimorbidity patterns, especially the arthritis-cataract group (OR: 1.70, 95% confidence interval [CI]: 1.02–2.82) and the multimorbidity group (OR: 2.30, 95% CI: 1.50–3.54), were linked with a significantly increased incident depression after 16 years. Additionally, being female, poor and fair income satisfaction, no social participation, poor and fair self-rated health, no alcohol consumption and admission during the past year was also significantly associated with incident depression.

**Table 2:** Univariate logistic regression of demographic and clinical characteristics predicting depression

		Depression		*P-value*
		OR	95% CI	
Disease patterns				
	Cardiometabolic	1.611	0.933–2.782	0.0869
	Arthritis-cataract	1.695*	1.019–2.820	0.0421
	Relatively healthy	Ref		
	Multimorbidity	2.302*	1.497–3.541	0.0001
Age		1.010	0.993–1.027	0.2692
Sex				
	Male	Ref		
	Female	1.777*	1.367–2.311	< 0.0001
Income satisfaction				
	Poor	1.694*	1.126–2.549	0.0115
	Fair	1.394*	1.045–1.859	0.024
	Good	Ref		
Social participation				
	Yes	Ref		
	No	1.584*	1.219–2.057	0.0006
Self-rated health				
	Poor	2.972*	2.110–4.186	< 0.0001
	Fair	1.637*	1.203–2.227	0.0017
	Good	Ref		
Smoking				
	Yes	0.776	0.565–1.067	0.119
	No	Ref		
Alcohol consumption				
	Yes	0.704*	0.510–0.971	0.0326
	No	Ref		
Betel nut chewing				
	Yes	0.8	0.468–1.369	0.4153
	No	Ref		
Admission in the past year				
	Yes	1.634*	1.117–2.391	0.0114
	No	Ref		
Exercise				
	No	0.81	0.521–1.259	0.3485
	≦ 2times/week	1.007	0.544–1.862	0.9825
	3–5 times/week	Ref		
	≧ 6times/Week	0.698	0.444–1.098	0.1198
Disability				
	Yes	1.259	0.669–2.371	0.4751
	No	Ref		

**Abbreviations**: OR, odds ratio; CI, confidence interval.

Table 3 demonstrates the multivariable logistic regression results. Participants in the multimorbidity group had an increased risk of incident depression (OR: 1.62, 95% CI: 1.02–2.58) after adjusting for gender, social participation, alcohol consumption, income satisfaction, self-rated health and admission in the past year. Test of interaction between distinct multimorbidity patterns and social participation showed there was no interaction between each multimorbidity patterns and social participation (Supplementary Table 1).

**Table 3:** Multivariable logistic regression of demographic and clinical characteristics predicting depression

		Depression		
		OR	95%CI	*P-value*
Disease patterns				
	Cardiometabolic	1.33	0.745–2.375	0.3345
	Arthritis-cataract	1.23	0.704–2.147	0.4675
	Relatively healthy	Ref		
	Multimorbidity	1.623*	1.019–2.584	0.0413
Sex				
	Male	Ref		
	Female	1.607*	1.184–2.182	0.0023
Social participation				
	Yes	Ref		
	No	1.225	0.922–1.626	0.1611
Alcohol consumption				
	Yes	0.996	0.686–1.445	0.9816
	No	Ref		
Self-rated health				
	Poor	2.093*	1.434–3.054	0.0001
	Fair	1.369	0.995–1.883	0.054
	Good	Ref		
Income satisfaction				
	Poor	1.574*	1.030–2.405	0.0361
	Fair	1.286	0.954–1.733	0.0986
	Good	Ref		
Admission in the past year				
	Yes	1.295	0.856–1.960	0.2215
	No	Ref		

^*^, Significant (*p-value* < 0.05)

**Abbreviations**: OR, odds ratio; CI, confidence interval.

Table 4 shows the association between multimorbidity patterns and incident depression among participants with and without social participation. Compared to participants in the relatively healthy group with social participation, participants in the arthritis-cataract group without social participation (OR: 2.22, 95% CI: 1.03–4.78) and the multimorbidity group without social participation (OR: 2.21, 95% CI: 1.14–4.30) had significantly increased risk of having depression after adjusting for gender, self-rated health, income satisfaction, alcohol consumption, admission in the past year.

**Table 4:** Association of social participation with distinct multimorbidity patterns in the relationship of depression

		Multimorbidity Patterns			
		Cardiometabolic	Arthritis-cataract	Relatively healthy	Multimorbidity
With social participation (1300)		1.717 (0.820–3.592)	0.817 (0.357–1.869)	Ref	1.410 (0.750–2.649)
Without social participation (675)		1.098 (0.437–2.759)	2.218* (1.030–4.778)	1.159 (0.837–1.605)	2.211* (1.138–4.296)

Model was adjusted with: age, gender, self-rated health, income satisfaction, admission in the past year, alcohol consumption

Data are presented as odds ratios with confidence intervals (CI)

^*^, Significant (*p-value* < 0.05).

## Discussion

LCA discovered four distinct multimorbidity groups in this 16-year population-based longitudinal study among older adults in Taiwan, including the cardiometabolic, Arthritis-cataract, multimorbidity and relatively healthy groups. Additionally, we found that older adults in the multimorbidity group had a greater risk of developing depression than the relatively healthy group after the multivariable logistic regression with adjustment for potential confounding factors.

There was few evidence regarding the association of multimorbidity or multimorbidity patterns with incident depression. Notably, our results were different from those of the previous study. Hsu and Hsu using also TLSA to analyse the trajectory of depressive symptoms among older adults with different chronic diseases and found that older adults with cardiovascular disease (CVD), gastrointestinal disease, chronic respiratory disease (CRD), and the combination of two of these three diseases had a greater effect on the intercept of depressive symptoms. Only the older adults with CRD combined with CVD or with GI disease had a significant negative effect on the slope of depressive symptoms [38]. They used different groups of multimorbidity and this could be the reason that our results were different. Yao et al. discovered four somatic multimorbidity patterns among older adults in China, including cardiometabolic, respiratory, arthritic-digestive-visual and hepatic-renal-skeletal patterns, with all multimorbidity patterns linked with a higher risk of having depressive symptoms after the 4-year follow-up. [15] The possible explanation for the different results is the different reference groups. Our previous work revealed that people in the relatively healthy group still had several chronic diseases, such as arthritis. [16] Another possible reason is the statistical method used to locate the multimorbidity patterns. Notably, the multimorbidity patterns vary depending on the method of analysis used, such as Hierarchical cluster analysis (HCA) or exploratory factor analysis (EFA). A previous comparative study found EFA was useful in describing comorbidity relationships, and HCA could be useful for an in-depth multimorbidity study. [39].

Several hypotheses of the mechanism between multimorbidity and depression exist, and the linkage may be bidirectional. [40] Additionally, for patients with multimorbidity, there was more care burden, [41] symptom burden, [42] disability, [43] poor quality of life, [44] poor self-rated health [45] and pain, [46] which all lead to depressive emotion. Depression-related poor self-care, [47] alcohol consumption [48] and suicidal ideation [49] could also lead to multimorbidity. Furthermore, evidence shows that the inflammation process was induced by multimorbidity and such inflammation could also become a risk factor for depression. [50] For the relationship between psychological burden and chronic diseases, a past study revealed that there is psychological burden at different times for different chronic conditions. In addition, minimizing the incidence of comorbidities, physical limitations, or psychiatric conditions may have the prospective effect of avoiding the trend of increased depressive symptoms. [51] Combine with our results, healthcare professional should put more emphasis on noticing possible depressive symptoms among older adults with multimorbidity burden.

Our results suggest that participants in the arthritis-cataract group without social participation and the multimorbidity group without social participation had significantly increased risk of having depression, compare with participants in the relatively healthy group with social participation. Social participation has proved to be related to less depression among the elderly in different populations. [52, 53] However, Galenkamp et al. demonstrated that older adults with multimorbidity had less social participation; higher socioeconomic status, widowhood, a larger network of friends, volunteering, transportation possibilities and fewer depressive symptoms were essential for social leisure participation. [54] Another study in the UK showed that physical multimorbidity reduced some aspects of social participation over time. [55] For the relationship between social participation and multimorbidity, previous study showed that older adults with higher emotional-social support had lower depressive symptoms, and the effect of this support could reduce depressive symptoms even more over time [38]. From our results, social participation seems to be a protective factor against incident depressive symptoms, especially among participants in arthritis-cataract group and multimorbidity group. Moreover, participants of arthritis-cataract group without social participation seemed to have much difference compared with those who with social participation. Interaction between social participation and arthritis-cataract may exist. Therefore, future study should focus on the interaction and intervention of social participation among these groups of older adults and measure the outcomes. Also, from our results, it seems that there is no benefit of social participation among participants with cardiometabolic multimorbidity. One hypothesis is there are more factors influencing the relationship between cardiometabolic multimorbidity and social participation. Previous study showed that there are multiple factors contributing to social participation among diabetes patients, including self-management of treatment, lifestyle, mobility, subjective assessment of health, and quality of life. [56] There is also a Japanese report that social participation is associated with prevention of hypertension, and physical activity may play as a connective role. [57] Further study is suggested with considering these factors among larger population.

Several strengths exist in this study. Notably, this is the first longitudinal study of how social participation affects late life depression among participants with different multimorbidity patterns in an Asian country. The 16-year retrospective cohort study was based on a representative national sample with high survey response rates. Data from a large and randomly selected population have high external validity. Finally, we adjusted for numerous confounding factors, including gender, social participation, self-rated health, health behaviours and recent admission.

Our study also has several limitations. First, multimorbidity patterns can change over time. For example, older adults may develop more chronic diseases as they age. Therefore, the relationship between the multimorbidity group and depression could have been underestimated. However, our previous work examined the trend of multimorbidity patterns for 16 years in a similar population and found that disease patterns remained similar in five waves of data (Supplementary Figs. 1–5). [16] We then proposed that the composition of disease groups was similar in subsequent years. Future study can be done using latent transition analysis (LTA) or group-base trajectory analysis [58] to include the multiple waves of multimorbidity. Second, self-reported data were used from TLSA without objective measurements, resulting in reporting bias. Third, the depression was related to poor cognitive performance. However, no relative information regarding cognition could be obtained in 1996 from TLSA. Future studies should explore the relationship between cognitive performance and depression in patients with different multimorbidity patterns. Third, we used data from 1996 to 2011, and the types and methods of social participation could change over time. We would like to conduct further study regarding multimorbidity patterns and social participation in the future with newest data. Fourth, because the characteristics of longitudinal study, the possible selection bias by death or loss follow-up of the participants could still happen.

## Conclusion

This 16-year population-based cohort study revealed that distinct multimorbidity patterns among older adults in Taiwan were associated with incident depression during later life. However, social participation can be a protective factor against future depression among older adults with different multimorbidity patterns. Therefore, further studies should focus on identifying multimorbidity and timely intervention of social participation encouragement.

## Electronic supplementary material

Below is the link to the electronic supplementary material.

Supplementary Material 1. Supplementary Figure 1. Multimorbidity patterns in 1996. Supplementary Figure 2. Multimorbidity patterns in 1999. Supplementary Figure 3. Multimorbidity patterns in 2003. Supplementary Figure 4. Multimorbidity patterns in 2007. Supplementary Figure 5. Multimorbidity patterns in 2011.

Supplementary Material 2. Supplementary Table 1. Multivariable logistic regression of demographic and clinical characteristics predicting depression with interaction of multimorbidity patterns and social participation.

## Data Availability

The datasets used and analysed during the current study are not publicly available, but are available from the corresponding author on reasonable request with the permission of the Ministry of Health and Welfare, Taiwan.

## List of Abbreviations

YLD: Years Lived with Disability.
TLSA: Taiwan Longitudinal Study on Aging.
ADL: Activities of Daily Living.
LCA: Latent Class Analysis.
EFA: Exploratory Factor Analysis.
HCA: Hierarchical Cluster Analysis.
