# Supplementary material for: Association between multimorbidity patterns and incident depression among older adults in Taiwan: the role of social participation
Source: BMC Geriatr. 2023 Mar 27;23:177. doi: 10.1186/s12877-023-03868-4 (PMC10045862; doi:10.1186/s12877-023-03868-4)
Supplement: Supplementary file 1 — Supplementary Material 1. Supplementary Figure 1. Multimorbidity patterns in 1996. Supplementary Figure 2. Multimorbidity patterns in 1999. Supplementary Figure 3. Multimorbidity patterns in 2003. Supplementary Figure 4. Multimorbidity patterns in 2007. Supplementary Figure 5. Multimorbidity patterns in 2011. [file 12877_2023_3868_MOESM1_ESM.docx]

**Supplementary Figure**

**Supplementary Figure 1.** Multimorbidity patterns in 1996.

**Supplementary Figure 2.** Multimorbidity patterns in 1999.

**Supplementary Figure 3.** Multimorbidity patterns in 2003.

**Supplementary Figure 4.** Multimorbidity patterns in 2007.

**Supplementary Figure 5.** Multimorbidity patterns in 2011.

**References:**

1. *Ho HE, Yeh CJ, Wei JC, Chu WM, Lee MC:* ***Trends of Multimorbidity Patterns over 16 Years in Older Taiwanese People and Their Relationship to Mortality****. International journal of environmental research and public health 2022,* ***19****(6).*
