# Supplementary material for: Association between multimorbidity patterns and incident depression among older adults in Taiwan: the role of social participation
Source: BMC Geriatr. 2023 Mar 27;23:177. doi: 10.1186/s12877-023-03868-4 (PMC10045862; doi:10.1186/s12877-023-03868-4)
Supplement: Supplementary file 2 — Supplementary Material 2. Supplementary Table 1. Multivariable logistic regression of demographic and clinical characteristics predicting depression with interaction of multimorbidity patterns and social participation. [file 12877_2023_3868_MOESM2_ESM.docx]

Supplementary Table

Supplementary Table 1. Multivariable logistic regression of demographic and clinical characteristics predicting depression with interaction of multimorbidity patterns and social participation

|  | | | | |
| --- | --- | --- | --- | --- |
|  |  | Depression | | |
|  |  | OR | 95%CI | *P-value* |
| Disease patterns | |  |  |  |
|  | Cardiometabolic | 2.021 | 0.636–6.423 | 0.233 |
|  | Arthritis-cataract | 1.056 | 0.418–2.666 | 0.908 |
|  | Relatively healthy | Ref |  |  |
|  | Multimorbidity | 2.015 | 0.681–5.962 | 0.206 |
| Sex |  |  |  |  |
|  | Male | Ref |  |  |
|  | Female | 1.608* | 1.182–2.189 | 0.003 |
| Social participation | |  |  |  |
|  | Yes | Ref |  |  |
|  | No | 1.564 | 0.500-4.891 | 0.442 |
| Disease patterns x social participation | |  |  |  |
|  | Cardiometabolic x social participation | 0.235 | 0.049-1.136 | 0.072 |
|  | Arthritis-cataract x social participation | 0.552 | 0.169-1.798 | 0.324 |
|  | Relatively healthy x social participation | Ref |  |  |
|  | Multimorbidity x social participation | 0.408 | 0.099-1.683 | 0.215 |
| Alcohol consumption |  |  |  |  |
|  | Yes | 0.998 | 0.686–1.450 | 0.990 |
|  | No | Ref |  |  |
| Self-rated health | |  |  |  |
|  | Poor | Ref | 1.481–3.141 | <0.0001 |
|  | Fair | 0.645* | 0.452–0.921 | 0.016 |
|  | Good | 0.467* | 0.320-0.683 | <0.0001 |
| Income satisfaction | |  |  |  |
|  | Poor | Ref | 1.040–2.426 | <0.0001 |
|  | Fair | 0.821 | 0.548–1.232 | 0.341 |
|  | Good | 0.629* | 0.411-0.962 | 0.033 |
| Admission in the past year |  |  |  |  |
|  | Yes | 1.298 | 0.856–1.967 | 0.219 |
|  | No | Ref |  |  |
|  |  |  |  |  |

^*^, Significant (*p-value* < 0.05)

**Abbreviations:** OR, odds ratio; CI, confidence interval.
